# Involving a Citizens’ Jury in Decisions on Individual Screening for Prostate Cancer

**DOI:** 10.1371/journal.pone.0143176

**Published:** 2016-01-11

**Authors:** Paola Mosconi, Cinzia Colombo, Roberto Satolli, Sara Carzaniga, Steering Committee and the Scientific Committee

**Affiliations:** 1 Laboratory for medical research and consumer involvement, IRCCS Istituto di Ricerche Farmacologiche Mario Negri, Milano, Italy; 2 Agenzia di Editoria Scientifica Zadig, Milano, Italy; 3 Agenzia Nazionale per i servizi Sanitari regionali Agenas, Roma, Italy; ISPO, ITALY

## Abstract

**Aims:**

Most public health agencies and learned societies agree that the prostate-specific antigen (PSA) test in asymptomatic men should not be recommended, on account of its potential for harm. Yet PSA is still widely used as a screening test and is not being abandoned. This remains a significant public health issue, and citizens’ engagement is needed. This study was designed to produce a deliberation on the PSA screening test by a citizens’ jury.

**Methods:**

Fifteen citizens were selected and balanced for sex, age, and education. They received an information booklet and participated in a two-day meeting with experts to reach a deliberation on the question “Should the National Health Service discourage or recommend PSA as an individual screening test for prostate cancer in men 55–69 years old?”. A facilitator ran the jurors’ discussion.

**Results:**

All except three of the jurors decided that the National Health Service should discourage the use of PSA as an individual screening test for prostate cancer in 55–69 year-old men. The jury was particularly convinced by the uncertainty of the test outcomes, the utility of the test, and its cost/benefit ratio. Before the meeting 60% of jurors would have recommended the test to a relative, and all the male jurors would have done so. After the meeting these percentages fell to 15% and 12%.

**Conclusions:**

This experience confirms the feasibility and effectiveness of delegating to a group of citizens the responsibility to decide on public health issues on behalf of the community. Public health authorities should invest in information campaigns aimed at the public and in educational initiatives for physicians. This also provided an opportunity to disseminate information on screening, over-diagnosis, and over-treatment.

## Introduction

Prostate cancer is one of the most widely diagnosed malignancies among men in developed countries. In Italy, about 36,000 new cases and about 7,800 deaths were recorded in 2012 [1]. The benefits and harms of the prostate-specific antigen (PSA) screening test to reduce specific mortality have been debated for a long time.A recent Cochrane systematic review [2], combining data from five randomized clinical trials (RCT) with more than 340,000 participants, including two important recent RCTs in Europe [3] and USA [4], found that PSA screening did not reduce the specific mortality for prostate cancer, and tended to lead to over-diagnosis and consequent over-treatment. Similar conclusions have been drawn by two other reviews [5,6], one showing a small or no reduction [6], the other no significant effect [5] on prostate cancer-specific mortality A recently population-based cohort study showed a possible greater benefit in high-risk men aged 60 with PSA ≥ 2 ng/mL [7]. Nevertheless, in view of the nature of the study, this finding must be taken with caution.

Most clinicians, [8–10] learned societies [11,12] and public health committees [13,14], as the US Preventive Service Task Force and the UK National Screening Committee, now agree that the PSA test should not be recommended as screening for prostate cancer in asymptomatic men [15]. At the individual level the PSA decision should be based on a clear understanding of the benefits and harms, with respect for personal values, in the framework of shared decision-making [16], as strongly recommended by scientific societies, institutional and independent groups [12–14].

The discrepancy between the widespread use of the PSA test in clinical practice and the recommendations of several international guidelines against it is widely debated [17,18]. The suggestion for judicious use of PSA remains largely unheeded [12,19,20], and the PSA incidence rates rise year by year, particularly in some categories of men. In Italy, about half the men over fifty had at least one PSA test [21]. The National Health Service (NHS) published a summary of the evidence [22], leaving each Regional Health Service free to decide how to implement it. The PSA test, as a screening tool, therefore remains a significant public health issue, and not surprisingly it is on the American Academy of Family Physicians’ list of the 15 things physicians and patients should question, according to Choosing Wisely [20].

This screening could be viewed in the context of the debate on the increasing “medicalization” of society [23] where the estimated benefits may be overwhelmed by the drawbacks (over-diagnosis, over-treatment) in public health, social and economic terms. As the benefits and harms of screening involve the whole community, but are hard to assess at the individual and community levels, and PSA opportunistic screening is a controversial issue this debate cannot be solved solely on the basis of scientific evidence or expert opinions, without consulting the general population for its preferences and values [24]. A different approach to establishing public involvement is required [24].

The Citizens’ jury is a method of deliberative democracy where a group of lay citizens with different backgrounds, values and attitudes is given adequate and independent information, then deliberates on an issue of interest to the community [25,26].

This article reports the results of a Citizens’ jury on the PSA individual screening test for prostate cancer.

## Methods

This project was coordinated by researchers involved in community empowerment initiatives [27], in collaboration with science communication experts and clinicians. The promoters were IRCCS Istituto di Ricerche Farmacologiche Mario Negri, Agenzia di Editoria Scientifica Zadig, and Agenas—the Italian National Agency for regional health services [28]. The process was supervised by a multidisciplinary steering committee, where advocate groups of patients were involved. According to Italian law, ethical approval is not required for this kind of study. The jurors, after receiving a leaflet giving information about the project, signed a letter of agreement to participate, and consented to the use of their data for research purposes.

### The questions for the jury

The main question, discussed and agreed among promoters and the steering committee, was: “Should the NHS discourage or recommend PSA as an individual screening test for prostate cancer in 55–69 years old men?”. The question was formulated in the name of the NHS with the aim to making the jury deliberate from the point of view of the common good—not the individual good.

The main question was accompanied by four sub-questions.

### Selection of the jury

The jury was selected among the members of the “Laboratorio dei cittadini competenti di Modena” (Laboratory of competent citizens of Modena), a voluntary group of lay adults with no specific competence in health/medicine issues, active in support of institutional health information campaigns [29].

Men and women over 18 years old, with no personal or family history of prostate cancer—so as not to deliberate as patients—were eligible. Among those willing to participate, a group of 15 members balanced for sex, age and education was selected. Each member received a fee of 100 Euros.

### The information for the jury

An *ad hoc* booklet was prepared on the basis of a review of the literature. To collect any pertinent document, a public call was launched on the PartecipaSalute website [27]; learned societies, patients’ and consumers’ associations and public health offices were directly invited by e-mail. The 25 documents submitted were examined. A sample of consumer/patient organizations’ websites was also visited to catch topics of interest. The draft of the booklet was discussed by the promoters, the steering group and with the PartecipaSalute-GRAL, a group of patients and consumers’ representatives [27]. The topics of the 30-page booklet are reported in Appendix A.

Nine experts were invited to the two-day meeting with jurors: four epidemiologists—some of them part of a national screening group—a urologist, a general practitioner, an oncologist, a health policy maker, and an expert in health economics. Topics covered are reported in Appendix A. Interviews with three middle-aged men were presented in videos (Appendix A).

### The two-day meeting and the deliberation session

The first and second days the experts gave their talks and discussed with the jurors. A final debate was organized between a clinician and a health policy maker on the pros and cons of opportunistic PSA screening. In the afternoon of the second day, a four-hour closed-doors session was dedicated to the discussion among the jurors to deliberate. Before the session two participants, for strictly personal reasons, decided to leave the Citizens’ jury. The discussion was assisted by a psychologist expert in group facilitation, to create a favourable climate for jurors, to share their points of view freely, and discuss amongst themselves until they reached a consensus, if possible. The facilitator used an argumentative style for conducting the meeting.

One representative of the jurors was responsible for drafting the deliberation, presenting it to the experts and promoters at the end of the second day, and writing the final document in collaboration with the facilitator. The final document, describing the deliberation and its reasons, was circulated, amended and approved by all the jurors.

### The questionnaires for the jury

The jurors were asked to complete two self-administered questionnaires.

The first was answered before the start of the jury meeting, the second at the end. The first comprised ten closed questions on knowledge of prostate cancer and the Citizen’s jury method, on the quality of the information booklet, on the individual’s attitude toward PSA testing, and on additional information seen before the meeting. The second comprised 13 closed questions on the quality of the experts’ presentations, the time dedicated to the jury discussion meeting, the role of the facilitator, the attitude toward PSA testing, and opinions about the Citizen’s jury.

To assess the changes before and after the jury, some questions were repeated in both questionnaires. Free comments were also collected.

Descriptive statistics, mainly proportions, were used to analyze all data collected. Data were collected and analyzed using Microsoft Excel.

To collect feedback also from the experts involved in the project, the promoters asked them to share their opinions about the strengths and limits of this experience by e-mail. Comments were analyzed by promoters, and discussed in a meeting with the experts.

## Results

Fifteen jurors participated, two thirds were male (67%), with middle and high school education (73%), and mean age 58 years.

All except three of the jurors decided that the NHS should advise against the use of PSA as an individual screening test for prostate cancer in 55-69-year-old men. The reasons are collected in a deliberation literally reported in Box 1. The answers to the four sub-questions are reported in Appendix B.

**Box 1. The jury’s deliberation and its reasons**

The Citizens’ jury is aware of the importance of the problem, as prostate cancer is the most common cancer among men, directly affecting 36,000 people each year. The jury is also aware that the demand for several million PSA tests per year indicates a widespread need for reassurance. However, although PSA is the only available test for early diagnosis of prostate cancer, the majority of jurors—having consulted the experts and drawn their own conclusions—agree that currently the NHS should not recommend PSA as an individual screening test. The jury stresses that discouraging is not tantamount to banning, but expresses a general indication. In such a controversial field, this choice works in favour of the doctor's freedom without affecting the freedom of the citizens/patients. The recommendation does not prevent doctors prescribing PSA to an asymptomatic patient. However, it does protect a doctor who does not deem the test necessary, without fear of a lawsuit. It may also provide an opportunity for doctors to get further information about the test and the results of studies so far, and facilitates an open and honest discussion with the citizens who request the test. The Citizens’ jury motivates the recommendation with reasons concerning the uncertainty of the test outcomes, the individual and social utility of the test, and its cost/benefit ratio.

**Uncertainty of the test outcomes**

Like any diagnostic test, PSA has its own sensitivity and specificity, which the jurors do not consider optimal. In the case of a negative outcome, the test gives the patient a false sense of security, but many men with cancer have normal PSA levels (false negatives), and many with high PSA do not actually have cancer (false positives). This exposes a potentially very large number of men—more than half of those who have the test—to concerns and further tests, sometimes invasive such as a biopsy, which involve a certain risk.

**Individual and social utility**

In addition to false positives and false negatives, the test leads to over-diagnosis, i.e. the identification of cancers that would never have developed or that would have developed so slowly as to not significantly affect the person’s quality or quantity of life. Then too, if a cancer is diagnosed, the treatment may severely affect the quality of life of a large number of people, causing concern, urinary incontinence, erectile dysfunction, etc.

**Cost/benefit ratio**

The number of healthy individuals, or those with a slowly progressing cancer, who are likely to be treated unnecessarily, with consequences such as those described above, is considered too high in relation to the number of lives that early detection—in the best scenario—may actually save. This negative balance, in terms of harms for health, is further worsened when the costs of unnecessary treatments are added. These sums could be more usefully invested in information and research for more reliable tests.

The Citizen’s jury acknowledge that giving up on the idea of a test for early detection of such a widespread disease as prostate cancer is not easy. However, this test does not seem to be performing adequately and is likely to deceive or discourage a large number of people. Considering also the current limitations on resources, it is all the harder to justify recommending the test. The Citizens’ jury recommend that the outcomes of PSA tests done as individual screening in the 55-69-year-old group be carefully checked.

### The jurors’ discussion

During the meeting the jurors had lively discussions with experts and promoters. A couple of jurors was at first cautious about the aim of the project, asking for more information about the “real” interest of the promoters, as if they might have had a pre-set position about the jury’s outcome. The promoters—as underlined at the beginning of the meeting—reaffirmed their total neutrality, clarifying the aim of the project. Most of the jurors felt free to deliberate and personally responsible for the final deliberation.

During the closed-doors session, the discussion was introduced by asking the jurors to express reasons supporting the positions reported in the main question. The jurors’ reasons supporting the position “the NSH should recommend…” referred to the fact that prostate cancer is common, PSA tests are widely used, and “even if the PSA test is not perfect, it is the only one available”. Early diagnosis was considered a good thing in any case because “thanks to it the development of a cancer can be stopped”–not knowing *a priori* how it might develop over time. Another reason was personal autonomy: “a person has to be free to ask for the PSA test even if the doctor does not agree—to reduce anxiety (even considering the limits of the test) and to prevent the disease”.

The jurors’ reasons supporting “the NSH should discourage…” were mainly driven by the cost-benefit ratio, unfavorable due to the limits of the PSA test in terms of accuracy. The main issues considered were the severity of the adverse effects of the diagnostic process, the number of men treated or worried unnecessarily, compared with the uncertainty of the benefit in terms of lives saved.

The main consideration that settled the different views was that supporting the position “the NSH should discourage …” would foster the possibility of deciding freely to do a PSA test or not, both for general practitioners—relieving them of any defensive stand—and for citizens who would have access to balanced information from their general practitioners.

### The questionnaire results

All jurors rated this experience positively and most considered the Citizens’ jury method useful. Many jurors felt actually involved in a decision about a public health topic that matters to them, underlining the need to make the deliberation widely known.

Regarding the national screening programs, most jurors (93%) had participated in those organized locally (colon, breast and cervical cancer). Before the meeting, most of the jurors believed the PSA screening test was useful (71%) and would have recommended it to a relative (60%); all the men would have done so. After the meeting most jurors had changed their attitude (respectively 31%, 15%, and 12%).

Most judged the information provided good or satisfactory; only a few felt there was too much, and sometimes the language was too technical. A few jurors commented that they lacked the information to answer the question, commenting: “I would need more plain information”; “I wonder why the PSA test as for individual screening is so widely used in clinical practice considering the limits indicated in the scientific knowledge available…”. A selection of questionnaire responses is reported in Table 1.

**Table 1:** Questionnaires submitted before and after the jury met.

	BEFORE	AFTER
	Jurors 15	Jurors 13°
**Individual attitude towards the PSA test**		
Willingness to do the PSA test as opportunistic screening	10 (100%)^	1 (12%)*
**Information provided**		
The booklet: clarity and completeness of the information provided °°		
good	4 (28%)	
satisfactory	6 (43%)	
sufficient	4 (29%)	
insufficient	0	
Experts’ presentations: clarity and completeness of the information provided		
good		6 (46%)
satisfactory		3 (23%)
sufficient		4 (31%)
insufficient		0
Need for more information for the jury to answer		3 (23%)**
**The process**		
Time for discussion		
much		0
enough		9 (69%)
not enough		4 (31%)
The facilitator’s running of the meeting		
good		10 (77%)
sufficient		3 (23%)
insufficient		0
Opinion about the Citizens’ jury method		
positive		11 (85%)
neither positive nor negative		2 (15%)
negative		0

° two jurors left the jury for personal reasons

^responses refer to the 10 men selected for the jury

* responses refer to the 8 men in the jury

°° one response missing (14 responses in all)

**reasons provided in the Results section

### The experts’ feedback

The main issues identified were the representativeness of the jurors, the quality and balance of the information, the opportunity for the jurors to put questions and participate in the debate with the experts, the impact of deliberation. These points have been matched against the jurors’ feedback and commented in the discussion.

## Discussion

The Citizens’ jury deliberated to discourage PSA testing by the NHS as individual screening for prostate cancer in 55-69-year-old men. The deliberation was agreed even though most of the jurors individually had been favorable to PSA screening before the meeting. In particular, all the men jurors were in favor of the test, but this dropped to 12% after the meeting. It is important to note that the jurors were able to take the point of view of public interest, leaving aside their individual preferences, and to reach a shared deliberation. This might have been due to the construction of the question itself (asking about NHS), the selection criteria of the jurors, the explicit demand—made by the promoters during the two-day meeting—to take the point of view of the common interest, and the topics covered during the two-day meeting, referring to the public effects of opportunistic PSA screening.

This confirms that, given truthful, complete information backed by arguments and referenced sources, and sufficient time for discussion, lay people can deliberate on a complex, uncertain issue like prostate cancer screening from a public point of view [30,31,32]. The main items underlined by the experts and by some of the jurors agree with the four key criteria of Abelson [33] for judging the efficacy of a Citizens’ jury. We can look at them here point by point.

### Representativeness

The jurors were selected from a group of people calling themselves the “Laboratory of competent citizens”. This might be considered a limit for their representativeness, because they are not completely naive, may share information about specific healthcare topics, and may already be used to working in healthcare settings, and dealing with healthcare topics. However, the people in this citizens’ jury must be willing to deliberate for the community, and had represent no individual interest or stakeholders. For this reason we included men who did not have prostate cancer, and some women too. In addition, the jury members did not all know each other, and this sampling method was the most cost-effective in this project. Finally, the small number of participants—another issue relating to the representativeness—is a common feature of this kind of method [34].

### Procedure and processes

All the steps were carefully monitored, and transparency and sharing was guaranteed throughout the project. The promoters moderated the debates so as to foster the jurors’ participation, and get jurors involved in the discussion. A few jurors asked for more time to discuss with experts; most felt they were truly involved in a decision on a public health topic, reinforcing the sense of responsibility in their deliberation.

### The quality of information

It was an important challenge to provide balanced information on a topic on which scientific knowledge is almost unanimous (against it), but clinical practice takes another direction. To face this challenge, the jurors were exposed to information covering the systematic reviews [2,6]—and their limits; experts illustrating the possible future of the PSA test and the complexity of decision making in the clinical setting; videos of men satisfied with their decision to do the PSA test, and, finally, a debate about the pros and cons of the test as screening.

Involving lay people in the production and review of information material is a key strategy, also in the dissemination of scientific information to lay people, especially when asking for a deliberation.

### Outcome

The impact on healthcare policies was intended to be approached by disseminating the deliberation among the decision networks. Agenas sent it to the leading NHS institutions and to several pertinent learned societies. The deliberation was circulated to the general population through the media and discussed at a public meeting.

To boost its impact, we invited various learned societies to participate in the project. Although several members of the steering and the scientific committees were affiliated to different medical associations, the promoters did not obtain any official endorsement. Only one society of GPs fully participated in the project and spread the deliberation [35]. The scant participation of learned societies is a limit of the project.

## Conclusions

The authors were uncertain about what the jury would deliberate, but the result is similar to that issued by an Australian Community jury [30], conducted in the same period of 2013 in a very different cultural and social context, and with a quite different composition of the jury (only men 50–70 years old). Both juries deliberated that the national health service should provide general practitioners with a scheme to give high-quality, complete information on the PSA screening test.

It is interesting that the main difference is that the Italian jury deliberated to reach the general public as well, while the Australians did not. The Italian jurors believe that providing the public with complete information through a national campaign could foster awareness in a possible decision on PSA screening; while the Australian jurors believe it could cause unnecessary anxiety and alarm among men not interested in doing the test. To assess the generalizability of these opinions other juries need to be organized in different settings and countries—comparing the process, the information provided and the rationale for deliberation.

The jury’s deliberation agrees with most of the literature, learned societies, and public health orientation, but contrasts with clinical practice [9,11,13,14,20,36]. Evidence-based information campaigns and projects for health workers and the general population are needed to increase awareness about this kind of healthcare topics [37].

## Appendix A The Information for the Jury

### The sections of the booklet

- Description of the project and main question for deliberation
- What is the PSA test
- PSA test for prostate cancer screening—benefits and harms, estimate of costs, a table summarizing international and national guidelines
- General information about screening and over-diagnosis
- General information about prostate cancer, its incidence and prevalence in Italy
- What a citizen jury is and why it was organized on this topic
- Suggestions on how to find more information with links to web sites
- Glossary.

### Topics covered by experts during the two-day meeting

- Introduction to the project and the Jury method, the roles and responsibilities of individuals and society relating to health decisions
- What is prostate cancer, and the epidemiology
- What is PSA, the decision-making in the clinical setting
- What is the screening, scientific evidence on the PSA test and screening
- PSA: early diagnosis and opportunistic screening
- PSA: results on mortality
- PSA: overdiagnosis and overtreatment
- Economic impact of the PSA screening test.

During the two-day meeting speakers tried to use plain language, with easy-to-read slides, following a few simple instructions such as no acronyms, no technical terms without explanation, openness to answer all questions from the jurors, etc.

### Overview of the stories presented in the video interviews

All the men interviewed received an opportunistic PSA screening, ultrasound examination, and biopsy. One was given a low-risk diagnosis of cancer and agreed to follow an active surveillance program; the others had a negative result. All described the psychological impact of the clinical process: the first and second were very satisfied at having had the PSA test, the third was more critical. The videos of the two-day meeting are available at http://www.partecipasalute.it/cms_2/giurie-cittadini/prostata/2015.

All the steps and materials were available in a section of the website PartecipaSalute (*Giurie dei cittadini*: *screening per il cancro alla prostata*) http://www.partecipasalute.it/cms_2/giurie-cittadini/prostata.

## Appendix B

### What initiatives should the National Health Service employ to discourage or recommend PSA testing: recommendations, guidelines, awareness campaigns, information brochures, incentives or disincentives, other?

Awareness campaigns through the media (public service announcement) about the controversial results of the test.

Actively encouraging a healthy lifestyle as a form of prevention of prostate cancer through the written prescription of preventive measures, such as physical exercise.

Jurors were against asking a patient to sign informed consent before the PSA test because they believed, it would be of little value if not accompanied by good information. Jurors wanted to avoid citizens without an adequate basis for making a choice having to bear the burden of the decision.

Establishing that PSA tests done as individual screening for prostate cancer are to be paid by the citizen.

### To whom should the NHS initiatives be targeted: family doctors, specialists, patients' or citizens' associations, general public, other?

Awareness campaigns through the media and directly addressed to the public. Dissemination of information through meetings with citizens promoted by voluntary associations. Information campaigns aimed at general practitioners, through conferences and training courses. Information to urologists was not considered necessary as they were supposed to be already informed.

### What information should the NHS provide on PSA as a screening test and to whom should it be addressed?

Information should be provided on the uncertainty of the diagnosis made from the PSA test, false positives and false negatives. It is necessary to explain the consequences of over-diagnosis that may lead to deterioration in the patient's quality of life with no real benefits in terms of life expectancy. However, it is important that the information is complete, mentioning that the PSA test can save lives but that for every life saved dozens of people will face over-diagnosis and over-treatment and may suffer urinary incontinence and impotence. It is also useful to provide information on the lifestyles that have proved useful in reducing the incidence of prostate cancer such as not smoking, exercising, eating a balanced diet and so on.

### How should the quality and the independence from commercial or industry interests of the initiatives and information provided by the NHS be ensured?

Information should only be provided by public services concerned only with health. On informative tools no brands of pharmaceutical companies or manufacturers of devices or other medical products should appear.
